# Screening of hepatitis B and C viral infection, recognition of risk factors, and immunization of patients against hepatitis B virus: a module developed for effective hepatitis control

**DOI:** 10.3389/fpubh.2023.1269209

**Published:** 2023-11-23

**Authors:** Samina Ejaz, Iqra Abdullah, Waqas Nazir Malik, Shazia Anjum, Muhammad Ashraf, Naveed Akhtar, Aurangzeb Khan, Yasir Hameed, Muhammad Usman, Usman Cheema, Safeena Sidiq

**Affiliations:** ^1^Department of Biochemistry, Institute of Biochemistry, Biotechnology and Bioinformatics (IBBB), The Islamia University of Bahawalpur, Bahawalpur, Pakistan; ^2^Department of Biotechnology, Institute of Biochemistry, Biotechnology and Bioinformatics (IBBB), The Islamia University of Bahawalpur, Bahawalpur, Pakistan; ^3^Institute of Chemistry, The Islamia University of Bahawalpur, Bahawalpur, Pakistan; ^4^Department of Pharmaceutics, Faculty of Pharmacy, The Islamia University of Bahawalpur, Bahawalpur, Pakistan; ^5^District Head Quarter Hospital, Bahawalpur, Pakistan; ^6^Medical Division, The Islamia University of Bahawalpur, Bahawalpur, Pakistan; ^7^Women Health Care Center and Maternity Home (WHCC&MH), The Islamia University of Bahawalpur, Bahawalpur, Pakistan

**Keywords:** Hepatitis B virus, Hepatitis C virus, risk factors, screening, immunization and prevention

## Abstract

**Introduction:**

The continually increasing incidence of hepatitis, a worldwide health issue, in Pakistan, has highlighted the need to investigate the epidemiology factors and implement preventive measures accordingly. The purpose of this study was to scrutinize the prevalent and significantly associated risk factors of hepatitis in students and employees, screening them for hepatitis B and C virus and vaccinating them against HBV to make IUB hepatitis free.

**Methodology:**

A total of 12,912 participants including students (*n* = 10,948) and employees (*n* = 1964) were screened for HBV and HCV via immunochromatographic test. Hepatitis- positive participants’ blood samples were further tested and viral load was estimated by quantitative PCR. All the hepatitis-negative participants were vaccinated against HBV. The demographic and risk factors-related data were collected using the questionnaire. Statistical analysis (Chi-square test and bivariate regression analysis) was performed using SPSS software to explore any association between risk factors and hepatitis.

**Results:**

Results indicated that 662/12912 participants (students = 478/10,948, employees = 184/1,964) tested positive for hepatitis. Among them, HCV was observed to be more prevalent than HBV among the study participants, employees, and students, and viral count was low in both HBV and HCV-infected participants. However, men were more affected than women. The studied risk factors represented higher frequency among hepatitis-positive participants relative to the hepatitis-negative participants. The Chi-square test revealed that students’ gender, history of hepatitis in the family and relatives, dental treatment, sharing cosmetics and shaving blades were significant (*p* > 0.005) risk factors of hepatitis while in the employees group surgery and age were significant. Moreover, the reused of syringes was found to be associated with hepatitis in both groups. The bivariate analysis helped to identify various new risk factors which were independently, either positively or negatively, associated with hepatitis.

**Discussion:**

Our study enabled us to recognize different risk factors of hepatitis among the target population. The information thus generated can be usefully applied in planning hepatitis awareness, targeted screening, and effective control programs for other target populations. In general, this module can be further utilized for any other disease.

## 1 Introduction

Viral hepatitis is ranked the 8th leading cause of death around the world (1). This disease caused approximately 1.1 million mortalities around the globe in the year 2021 (2). Currently, a total of 257 million individuals (both men and women) are suffering from chronic hepatitis B and 71 million individuals (both men and women) are suffering from hepatitis C (3). If hepatitis keeps spreading at this rate, approximately 20 million deaths are expected to occur during 2020–2030 (1).

Hepatitis B virus is a member of the *Hepadnaviridae* family of animal viruses and carries a double-stranded genome 3.2 kb in length, while hepatitis C virus is classified in the *Hepacivirus* genus of the *Flaviviridae* family, and its genome is a positive-stranded RNA 9.6 kb in length (4). In terms of the global burden, Pakistan and Egypt are currently bearing an estimated 80% of the total hepatitis burden (5). Moreover, in Pakistan, currently, 12 million individuals (both men and women) are infected with hepatitis B or C (6) and this incidence is expected to increase in the coming years (6). Hepatitis disease is also known as a silent killer disease because most of the patients suffering from this disease remain undiagnosed and untreated for a long time before developing serious health complications (7). The major route of transmission of HBV is sexual contact, perinatal transmission, or horizontal transmission (8) while of HCV is infected blood (9).

Worldwide, the main risk factors for transmitting hepatitis B or C viruses are sexual contact, surgical procedures, skin tattoos, hemodialysis, being immune-compromised, household contact (10), reuse of syringes, surgery with contaminated instruments, blood transfusion, therapeutic injections, the use of unsterilized invasive medical devices, hospitalization, and sharing of razors (11). A few selective population groups are highly prone to be affected by hepatitis B and C, for example, drug users and thalassemia patients have more chances of getting hepatitis B and C infections (12).

In Pakistan, the identified major risk factors of HCV are having a HCV-infected mother, male sex, intravenous drug use, hospitalization, contaminated surgical equipment, circumcision by a barber, dental treatment (13) thalassemia, hemophilia, and blood transfusion (14). For HBV, the main factors are having a HBV-infected mother and circumcision by a barber (13), blood transfusion, reused syringes, surgery, hospitalization, shaving at a barber, needle injury, intravenous drug abuse, and sexual contact (15).

It is a fact that new cases of hepatitis infection are reduced in high-income countries (16), and the cases of this infection in many other low and middle-income countries, including Pakistan, are rising at a constant rate (17). Keeping in view the alarming situation regarding hepatitis infection, the government of Pakistan launched a national hepatitis sentry site observation system in June 2010 (Centers for Disease Control and Prevention 2011, CDC) to deal with the disease. However, due to the lack of proper infrastructure and the availability of facilities, this public health system was only limited to the regional capitals and Islamabad territory (18). Moreover, this program also restricted screening seropositivity in general people (18), excluding the high-risk groups like students studying in public sector universities. University students have not always been assumed as a priority while designing preventive and treatment policies because students are assumed to be healthier than others. However, during this time, students often adopt unhealthy lifestyles and habits, for example, eating poor foodstuffs, taking little rest, physical inactivity, smoking cigarettes, drinking alcohol, and drug abuse, which can badly affect their health in the short- or long-term (19, 20). Previous studies have highlighted that the lack of awareness among university students is one of the major Barrier to HCV treatment (21, 22), and early diagnosis can be a key milestone in hepatitis management (23).

Keeping in view the high prevalence of hepatitis disease in the Pakistani population, it was assumed that we could raise awareness about this disease in society by educating the students enrolled in Pakistani public sector universities. Therefore, the Vice Chancellor (VC) of IUB, Engineer Prof. Dr. Athar Mehboob, in collaboration with the ‘Hepatitis Control Program’ of the Health Department, Government of Punjab, Pakistan decided to launch a hepatitis control program at IUB. The purpose of this program was to raise awareness about hepatitis among the students and employees of IUB, screening them for hepatitis, vaccinating healthy individuals, and providing a facility of therapy for hepatitis-positive participants. Moreover, the program aimed to identify risk factors associated with the high prevalence of hepatitis in Pakistani students and employees. The ultimate goal of this program was to develop a module for the implementation of effective hepatitis control and ensure the status of IUB as a hepatitis-free zone. The vision of the hepatitis-free zone was to ascertain healthy youth which may finally lead to a healthy nation.

## 2 Methods

### 2.1 Program description and setting

**Figure 1:**
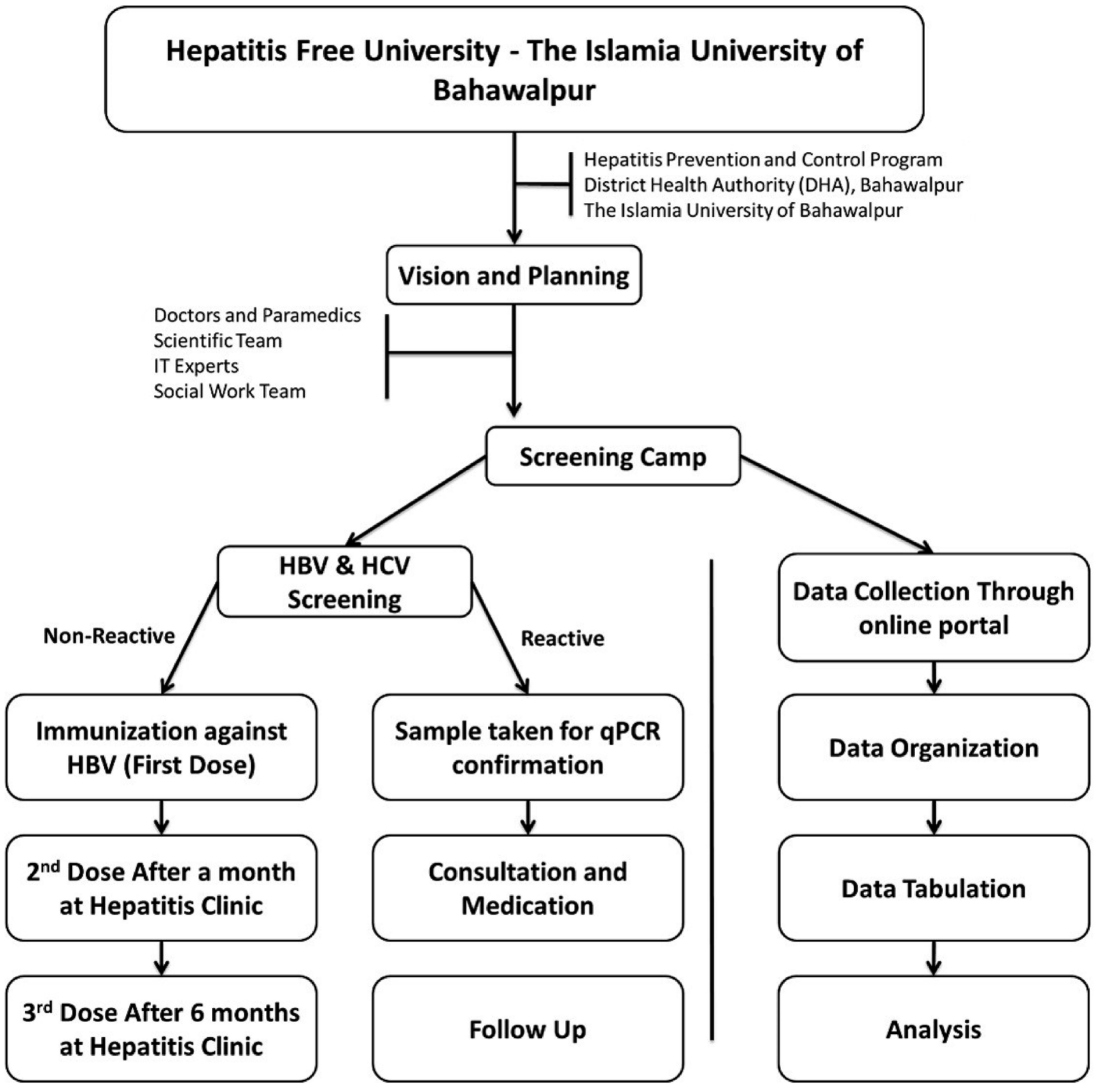
Overall work strategy. Represents the workflow of hepatitis control program including approval of program, sampling, data collection, and screening.

The present study was initiated in November 2019 and completed in February 2020. This study was designed as a joint venture of the District Health Authority (DHA), Bahawalpur, and IUB as an extension of the Hepatitis Prevention and Control Program (HPCP). The project was named “Hepatitis Free University-The Islamia University of Bahawalpur.” The work strategy is outlined in Figure 1.

### 2.2 Ethics approval

The presented study was approved by the IUB review board / ethical committee and was conducted following the guidelines of the Helsinki Model Convention 1992. All subjects were enrolled in the study after obtaining written informed consent individually from every participant, either student or employee.

### 2.3 Study design and planning

Different committees supervised by the focal person were constituted by the VC, IUB, and DHA, Bahawalpur for the smooth implementation of the program. The meetings of committee members were held regularly, and minutes of the meetings were written and circulated among the members for record-keeping and follow-up plans. The detailed plan for the implementation of this project was outlined with the consensus of all committee members.

#### 2.3.1 Public awareness plan

An extensive public awareness plan was chalked out comprising three layers. First, banners with the slogan “Hepatitis-Free University” were displayed all over the university and on the official social media pages of IUB and DHA. The DHA and IUB Medical & Health Division distributed brochures/awareness literature published by ‘Hepatitis & Infection Control Program Punjab’ among IUB students and employees. In all departments, the relevant literature was displayed on the noticeboard to ensure the passage of information to all stakeholders. Information on the “Hepatitis-Free University Program” was published in various local and national level newspapers by the PRO office of IUB. Various seminars were arranged 1 week prior to the execution of the plan at the faculty level and, finally, a mega seminar was arranged to sensitize the public regarding hepatitis-associated health hazards. Finally, SMS alerts were sent to the contact numbers of all students and employees to convey messages regarding the hepatitis-free university campaign.

#### 2.3.2 Policy for compliance

For 100% compliance, every student and employee was instructed to get screened and vaccinated as per convenience at the IUB screening camp or Punjab health care units/hospitals. It was notified that none of the students would be allowed to attend classes or participate in any activity without presenting their vaccination card. Similarly, employees were directed to comply with the orders to get due financial benefits.

#### 2.3.3 Scheduling of subjects for screening

Information about employees and students was obtained from the respective administrative offices, i.e., registrar’s office and respective departments. Specific days were assigned for each faculty’s students, teaching, and non-teaching staff for screening and vaccination. All the stakeholders were informed regarding the schedule and, according to the plan, participants were screened and vaccinated. For smooth implementation, chairpersons were directed to accompany students and staff on the screening camp and ensure their availability. To ensure 100% compliance, SMS alerts were sent to all scheduled subjects and all correspondence to chairpersons/heads was done through corresponding faculty deans and the registrar’s office, respectively.

### 2.4 Data collection and medical team

A medical team was recruited from the Health Department, Government of Punjab, and IUB Medical & Health Division for the screening and vaccination of participants. Dr. Usman Cheema, Senior Medical Officer (SMO) from the IUB medical division, and Dr. Aurangzeb, from District Head Quarters (DHQ) Hospital Bahawalpur, supervised the screening procedure, validated diagnostic/laboratory reports, and recommended vaccination. Moreover, Dr. Usman Cheema is responsible for supervising the treatment of hepatitis-positive patients.

For data collection, a team of volunteer students of BS Biochemistry and Biotechnology 6th semester (Session 2017–2021) and of the 8th semester (Session Spring 2016–2020) was trained.

### 2.5 Hepatitis screening

For hepatitis screening, a 26-day screening camp from 31st December 2019 to 25th January 2020 was held at the university. The screening camp was arranged at the university auditorium for the initial 20 days. Next, camps were held at the faculty level for 6 days. All the participants were recruited for screening according to their schedule during said duration (31st December 2019 to 25th January 2020).

In total, 12,912 participants including students (10,948) and employees (1964) were screened for HBsAg and anti-HCV. The Monolisa HBsAg Plus kit was used to screen hepatitis B, in human serum or plasma. The Hepatitis C screening was done with a Diasorian S. A kit.

### 2.6 Immunization

Non-reactive participants were immunized for hepatitis B virus. The first dose of the vaccine was given on the spot, the second dose after a month, and the third after 6 months. The vaccinations were completed at the hepatitis and infection control sentinel site established in IUB medical division BJ campus hospital.

### 2.7 PCR confirmation

Blood samples from HBV, HCV, or both positive participants were taken for confirmation of viral infection and viral load detection by qPCR. The samples were shifted to the central PCR section of the pathology laboratory in Bahawal Victoria Hospital (BVH), Bahawalpur for PCR confirmation. A viral count of >8,00,000 IU/mL was recorded as a high viral load, <8,00,000 IU/mL to be a low viral load, and < 20 IU/mL equals a very low viral load detected among hepatitis-positive participants.

### 2.8 Medication

The affected participants (having viral load) after the qPCR confirmation were called for consultation at the hepatitis clinic of the university’s hospital and Bahawal Vitoria Hospital Bahawalpur. The hepatitis-positive participants were given medicine according to the severity of the disease and the burden of viral load. The hepatitis-positive participants were followed up until they were cured to minimize complications, if any.

### 2.9 Data collection

A questionnaire was designed under the supervision of the scientific committee. It consists of three segments: (i) Demographic Information, (ii) Medical History, and (iii) Behavioral parameters. To make the data collection smooth and easy, the questionnaire was made available through an online portal. Each entry was saved at the local server at the university with the help of information technology (IT) experts of the university which ensured access to information of each participant during and after data collection. This helped in identifying individual participants. The screening test results and qPCR results were obtained from the team of DHA, Bahawalpur.

### 2.10 Studied variables

In the described study we have evaluated the following variables. Socioeconomic and demographic-related variables included Gender, Age, Income, Education, Marital status, Household contacts, Hepatitis history in relatives, Marriage trend, and Mother-infected with hepatitis. Medical-related variables were Blood group, Blood transfusion, Facial treatment, Surgery, Drug abuse/addiction, and already being vaccinated against HBV. Behavioral-related variables included smoking, reused syringes, and sharing shaving razors/machines, towels, and cosmetics.

Due to ethical conditions, some important worldwide known significant risk factors of HBV/HCV were not included in the present study, including transmission (promiscuity and homosexuality), alcohol consumption, and tattoos, as these are forbidden and are unlawful in our religion Islam.

### 2.11 Statistical data analysis

The data was split into two categories, employees and students, who were then processed separately for statistical analysis. The Statistical Package of Social Sciences (SPSS) software version 22 was used for the descriptive and bivariate statistics. For prevalence, the frequency of each parameter was calculated separately for the student and employee groups. The chi-square (*X*^2^) test was performed to determine the association between each risk factor (qualitative) and hepatitis in both groups. For *X*^2^, the alpha criterion was adjusted at 0.05 (95% confidence interval (CI)) and a *p*-value<0.05 was considered significant. For binary logistic regression “Multicollinearity assumptions test” was performed. Binary logistic regression using 95% CI (alpha criterion = 0.05) was employed to estimate the independent risk factors of hepatitis and the extent of the relationship between them in terms of the odd ratio of probability. The reference category was chosen to be the first.

## 3 Results

### 3.1 Success of execution plan

We have achieved the target of 100% screening and vaccination in two steps. At first in the mega camp (at university’s main auditorium), 60% of the IUB students and employees were screened and vaccinated against HBV. While for the remaining 40%, camps at the faculty level were arranged.

### 3.2 Socio-economic and demographic details of participants

Overall, 662/12912 (5.12%) subjects tested positive for hepatitis viral infection. Among them, 478/10,948 (4.3%) (male = 346/5539 (6.24%), female = 132/5409 (2.4%)) were student participants, while 184/1,964, i.e., 9.36% [male = 121/1216 (9.95%), female = 63/748 (8.34%)] were employees. Most hepatitis-positive students (59.21%) and hepatitis-negative students (85.58%) lie in the range of 16–25 years of age. While the higher ratio of hepatitis-positive and hepatitis-negative employees were noted to be in the 26–40 year age group (i.e., 30 and 37%, respectively). Most of the hepatitis-positive students (44.98%) and hepatitis-negative students (65.38%) were graduated (16 years of education). Among the employees, the majority, 30% (Hepatitis-positive) and 29% (Hepatitis-negative) of participants, had primary education (5 years of education). The highest frequency (32.62%) of hepatitis-positive students and 45.67% of hepatitis-negative students belong to the middle income (below 50,000) class and 26% of hepatitis-positive employees and 32% of hepatitis-negative employees were from low-income class. In our study, a large number (60.67%) of hepatitis-positive student participants and 91.41% of hepatitis-negative participants (students) were non-married while 6% of hepatitis-positive employees and 65% of hepatitis negative employees were married. Among them, an intrafamily marriage trend was observed in 68% of hepatitis-positive students participants and 53% of hepatitis-negative employees, while 25% of hepatitis-positive employee and 34% of hepatitis-negative employee reported interfamily marriages. Among students 4.8% of students of mothers who are hepatitis positive were also infected with hepatitis while 2.71% of students with hepatitis negative mothers were infected. Among employees, 1.08% with hepatitis positive mothers and 1.09% with hepatitis negative mothers were hepatitis positive (Table 1). The infection in any family member factor was found in only 17% of patients, 8% of hepatitis negative students, 7% of hepatitis positive employees, and 4% of hepatitis-negative employees. The Fisher exact test for household contact of hepatitis in students equals 0.01 and thus was associated with hepatitis. In 2% of hepatitis-positive student participants, infection history in their relatives was noted with an *X^2^* value of 8.6, *p*-value 0.00. The association of other socioeconomic factors with hepatitis has been shown in Supplementary Table S3.

**Table 1:** Frequency of demographic and socioeconomic parameters in hepatitis-negative and -positive participants.

Sr. No.	Factors	Response	Hepatitis negative students		Hepatitis positive students		Sr. No.	Factors	Response	Hepatitis negative employees		Hepatitis positive employees	
			Numbers	Frequency	Numbers	Frequency				Numbers	Frequency	Number	Frequency
1	Gender	Male	5,193	49.50%	346	72.38%	1	Gender	Male	1,095	61.50%	121	65.70%
		Female	5,277	50.40%	132	17.64%			Female	685	62.50%	63	34.20%
2	Age	16–25	8,960	85.58%	283	59.21%	2	Age	16–25	485	27%	16	9%
		26–40	871	8.32%	36	7.53%			26–40	662	37%	55	30%
		41–50	180	1.72%	11	2.30%			41–50	298	17%	31	17%
		Above 50	85	1%	3	1%			Above 50	218	12%	23	12%
		Not Answered	374	4%	145	30%			Not Answered	117	7%	59	32%
3	Education	Primary	107	1.02%	6	1.26%	3	Education	Primary	519	29%	55	30%
		Matric	106	1.01%	4	0.84%			Matric	342	19%	16	9%
		Intermediate	1,251	11.95%	36	7.53%			Intermediate	202	11%	6	3%
		Graduate	6,845	65.38%	215	44.98%			Graduate	245	14%	11	6%
		Post Graduate	1,585	15.14%	55	11.51%			Post Graduate	99	6%	5	3%
		Not Answered	576	5.50%	162	33.89%			Not Answered	376	21%	91	49%
4	Income	10,000–20,000	1,148	10.96%	54	11.30%	4	Income	10,000–20,000	566	32%	49	26%
		Above 20,000	1,169	11.17%	41	8.58%			Above 20,000	402	23%	38	21%
		Below 50,000	4,782	45.67%	156	32.64%			Below 50,000	497	28%	30	16%
		Above 50,000	2,806	26.80%	61	12.76%			Above 50,000	200	11%	8	4%
		Not Answered	565	5.40%	166	34.73%			Not Answered	118	7%	59	32%
5	Marital status	Single	9,571	91.41%	290	60.67%	5	Marital status	Single	629	35%	26	14%
		Married	899	8.59%	48	10.04%			Married	1,154	65%	109	59%
		Not Answered	0	0.00%	140	29.29%			Not Answered	0	0%	49	26%
6	Household contacts	Yes	824	8%	79	17%	6	Household contacts	Yes	78	4%	13	7%
		No	9,646	92%	399	83%			No	1705	96%	171	92%
		Not Answered	0	0%	0	0%			Not Answered	0	0%	0	0%
7	Infection history in relative	Yes	61	1%	8	2%	7	Infection history in relative	Yes	17	1%	2	1%
		No	10,409	99%	470	98%			No	5,176	290%	182	98%
		Not Answered	0	0%	0	0%			Not Answered	0	0%	0	0%
8	Marriage trend	Interfamily	4,455	43%	139	29%	8	Marriage trend	Interfamily	598	34%	46	25%
		Intrafamily	5,558	53%	325	68%			Intrafamily	1,032	58%	131	71%
		Not Answered	457	4%	14	3%			Not Answered	153	9%	7	4%
9	Mother-infected with hepatitis	Yes	284	2.71%	23	4.8%	9	Mother-infected with hepatitis	Yes	19	1.06%	2	1.08%
		No	10,186	97.2%	455	95.1%			No	1761	98.9%	182	98.9%

### 3.3 Distribution of hepatitis strains among participants

**Figure 2:**
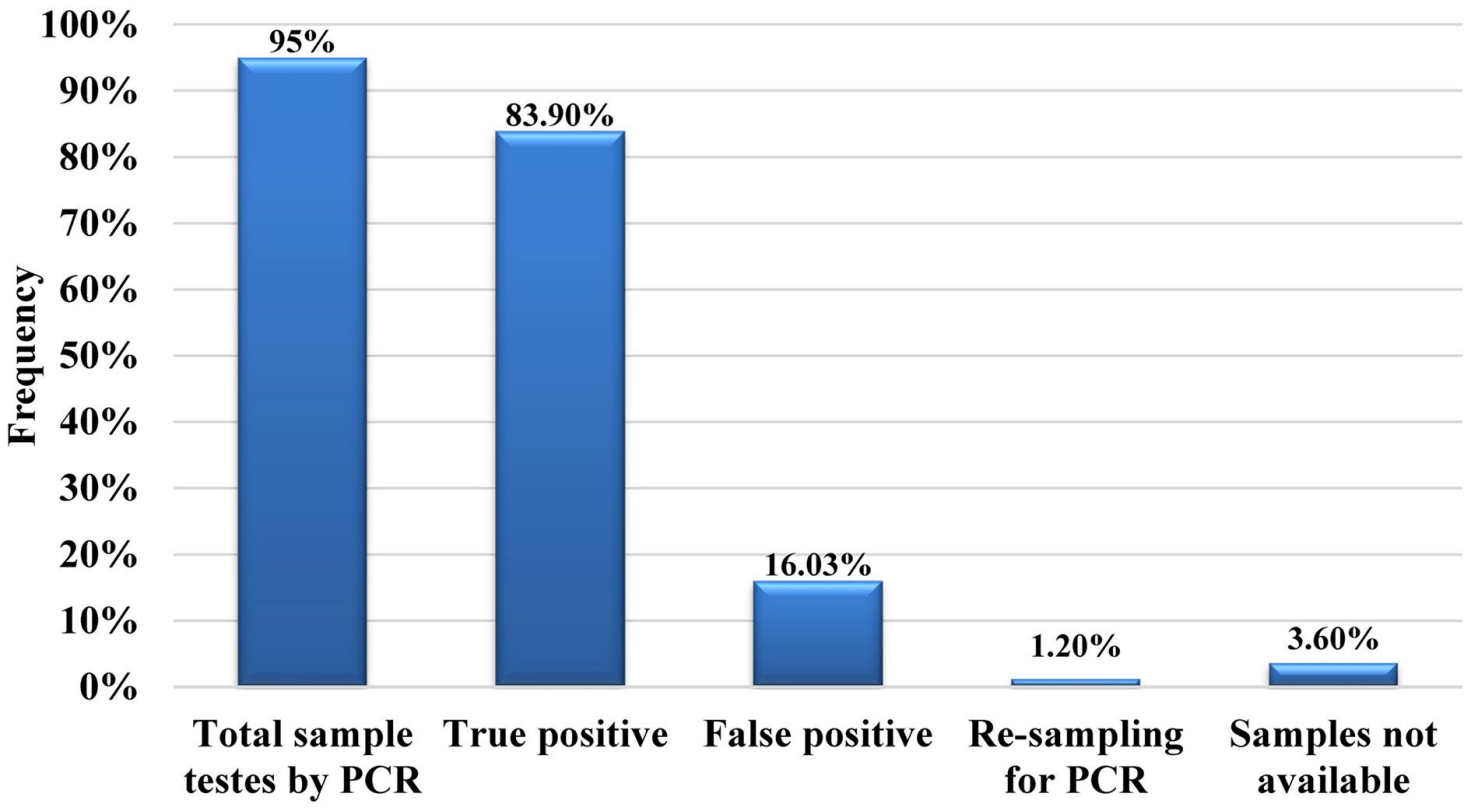
Hepatitis confirmation results by PCR. Represents the total samples for which PCR confirmation was done, the true positive and false positive percentage detected by PCR, re-sampling, and missing samples frequencies.

**Figure 3:**
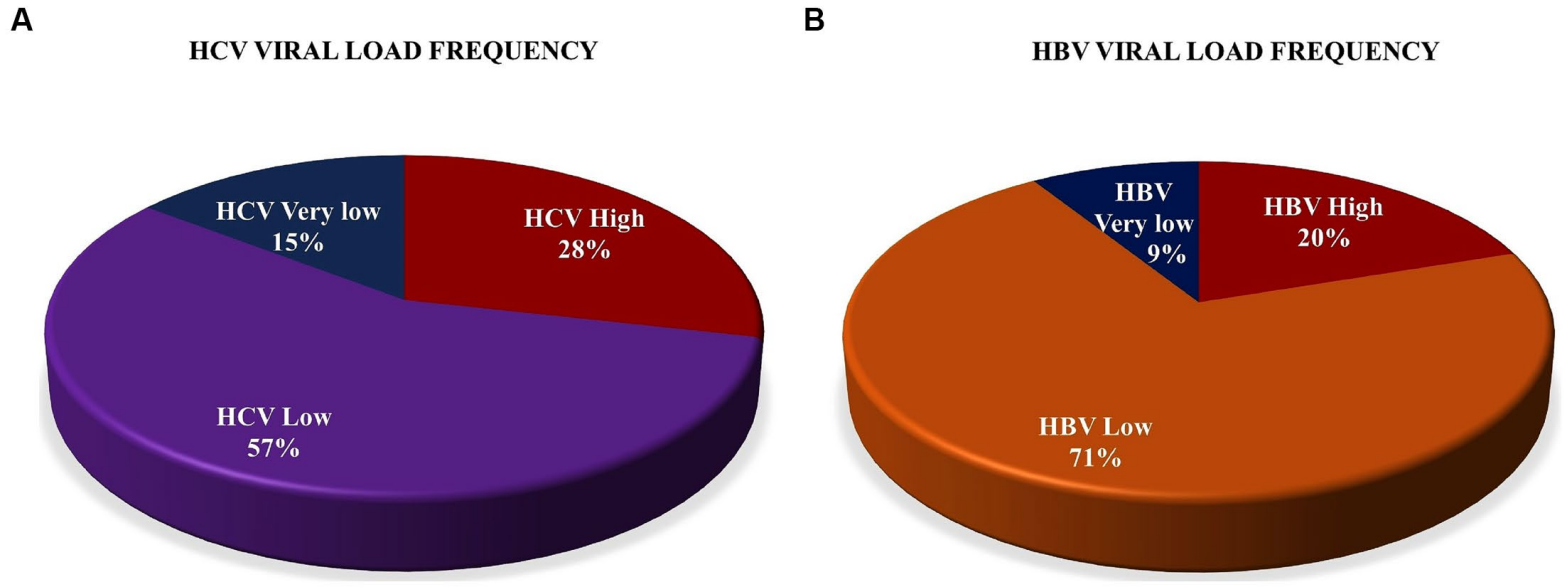
Hepatitis viral count. **(A)** HCV viral load frequency detected in hepatitis-positive participants. **(B)** HBV viral load frequency detected in hepatitis-positive participants. Viral count of >8,00,000 IU/mL recorded as high a viral load, <8,00,000 IU/mL to be a low viral load, and < 20 IU/mL equals a very low viral load detected among hepatitis-positive participants. HBV, Hepatitis B virus; HCV, Hepatitis C virus.

**Figure 4:**
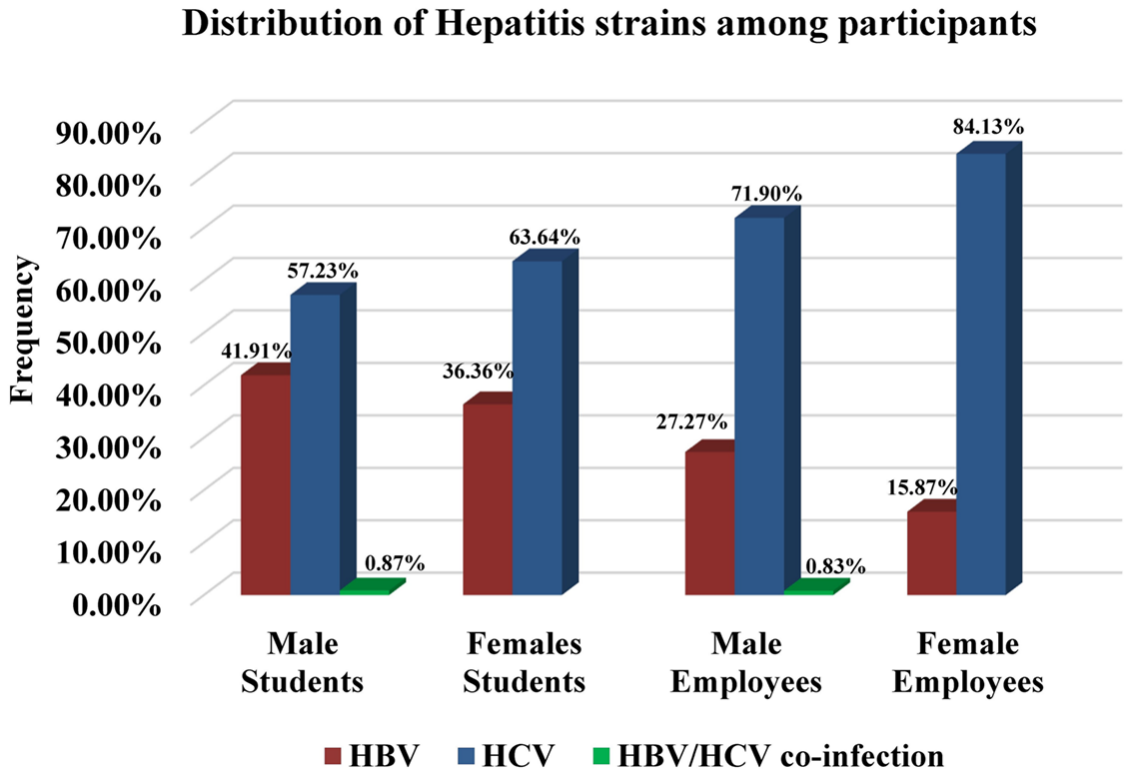
Distribution of hepatitis strains among hepatitis-positive participants. The prevalence of HBC, HBV, and co-infection of HBC and HBV was represented. HBV, Hepatitis B virus; HCV, Hepatitis C virus.

The 95% (630/662) detected (via immunochromatographic test (ICT) strip) hepatitis cases were tested further for PCR confirmation. Among them, 83.9% (529/630) were found to be true positive, i.e., positive results both by ICT strip and PCR and 16.03% (101/630) were noted to be false positive, i.e., positive results by ICT strip test while negative by PCR. For 1.2% (8/662), resampling was done, while 3.63% (24/662) samples of hepatitis-positive participants were not available (Figure 2). Viral count detection revealed a high viral load (Figure 3) in the majority of HCV-positive participants (57%) and a low HBV viral count in the majority of HBV positive participants (71%). The HCV was observed to be the most prevalent strain among hepatitis-positive participants with a frequency of 57.23% in male students, 63.64% in female students, 71.90% in male employees, and 84.13% in female employees. Among hepatitis positive individuals, a total of 41.91% of male students, 36.36% of female students, 27.27% of male employees, and 15.87% of female employee were confirmed to have HBV infection. There were only a few (0.87%) male students and 0.83% female students who tested positive for co-infection of HBV and HCV (Figure 4).

### 3.4 Prevalence and association of medical-related hepatitis risk factors

**Figure 5:**
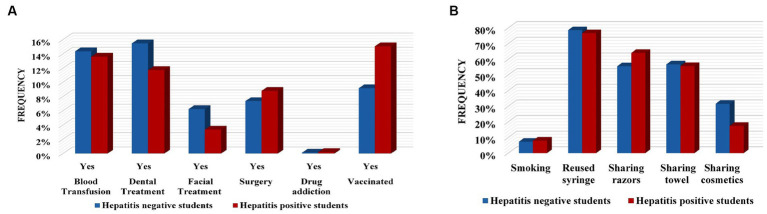
Frequency of various risk factors in hepatitis negative and positive students. **(A)** Frequencies of medical-associated factors. **(B)** Frequencies of behavioral-related factors.

**Figure 6:**
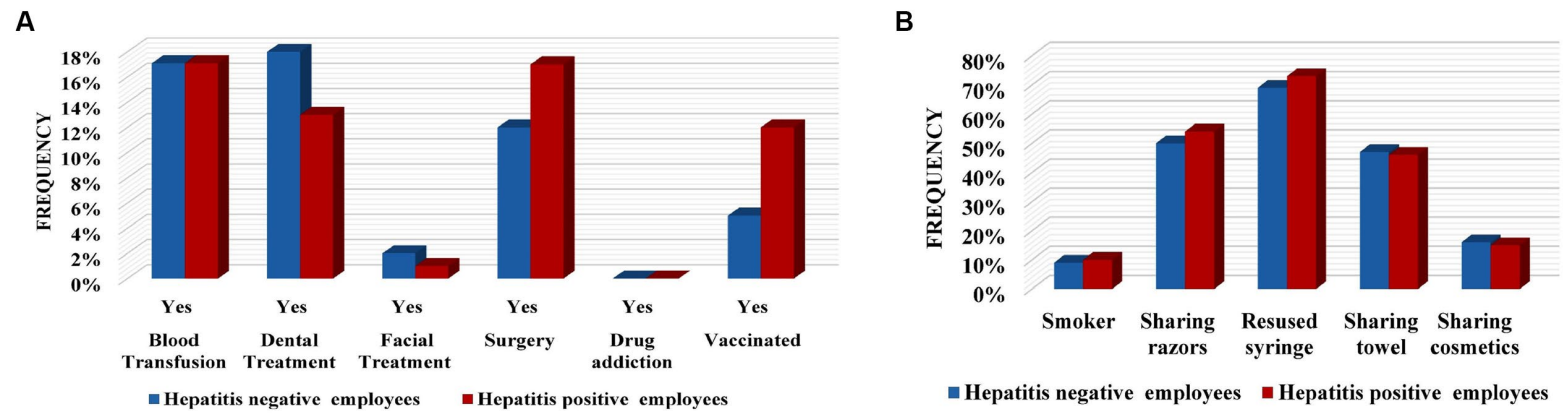
Frequency of various risk factors in hepatitis negative and positive employees. **(A)** Frequencies of medical-associated factors. **(B)** Frequencies of behavioral-related factors.

Evaluation of different risk factor frequencies and Pearson correlation measurements represent contrasting results. The frequency of some factors was found to be highest amongst hepatitis-negative participants. Frequencies of different blood groups varied among participants, but in hepatitis-positive and negative student participants, the B+ blood group was observed to be more prevalent (hepatitis positive students = 20%, hepatitis negative students = 24%, hepatitis-positive employee = 15%, and hepatitis-negative employee = 17%). The blood group was found to be not associated i.e., *p*-value 0.97 (students), *p*-value 0.08 (employees) with hepatitis. Another factor blood transfusion frequency was found to be 14% in hepatitis-negative students and 12% in hepatitis-positive students; 17% was detected in both the hepatitis-positive and negative employee participants. The *X^2^* shows no association of blood transfusion (*p*-value 0.79 in students, *p*-value 0.89 in employees) with hepatitis. Dental treatment frequency was found to be higher in the hepatitis-negative participants, with students at 15% and employees at 13%, while in the hepatitis-positive participants 12% was noted in students and 18% was noted in employees. The *X^2^* test reveals the relation of it with hepatitis as Pearson correlation value (3.694, *p*-value 0.05) was noted in the student group. More frequent facial treatments was observed among hepatitis-positive students (12%) than among hepatitis-negative students (6%). A very small number (1% hepatitis positive and 2% hepatitis negative) of participants (employees) were found to have facial treatment records and were found to be not linked (*p*-value 0.1 and *p*-value 0.23) with hepatitis. A total of 9% of hepatitis positive and 7% of hepatitis-negative participants (students) reported surgical history. Of employees, 17% of hepatitis-positive participants and 12% of hepatitis-negative participants reported having surgery in their life. An association of surgery (*X^2^* = 4.21, *p*-value 0.04) with hepatitis was shown by the employee group only. Neither the students nor employees were found to have a drug addiction and were not found to be associated (*p*-value 0.45 and *p*-value 0.78) with hepatitis in any group, respectively. We have observed that 15% of already vaccinated student participants and 12% of employee participants to be hepatitis positive while amongst hepatitis-negative participants only 8% of students and 5% of employees were observed to be already vaccinated. The association was found by the *X^2^* test with a measure of 21.09, *p*-value 0.00 in students and 13.2, and *p*-value 0.00 in the employees group (Figures 5, 6, Table 2, and Supplementary Tables S1, S2).

**Table 2:** Association of medical and behavioral risk factors with hepatitis.

Sr. No.	Risk factors	Groups	*X*^2^ values	*p*-value
A.	Medical related risk factors			
1	Blood group	Patients students	1.79	0.971
		Patients employees	12.56	0.083
2	Blood transfusion	Patients students	0.66	0.797
		Patients employees	0.17	0.897
3	Dental treatment	Patients students	3.694	0.05
		Patients employees	2.26	0.132
4	Facial treatment	Patients students	6.653	0.1
		Patients employees	1.39	0.238
5	Surgery	Patients students	1.36	0.243
		Patients employees	4.21	0.04
6	Drug addiction	Patients students	0.25	0.611 (Fisher value 0.465)
		Patients employees	0.103	0.748
7	Already vaccinated against hepatitis	Patients students	21.09	0
		Patients employees	13.2	0
		Patients employees	116.9	0.00
B.	Behavioral related risk factors			
1	Smoking	Patients students	0.345	0.559
		Patients employees	0.274	0.6
2	Avoid reused syringes	Patients students	45.16	0
		Patients employees	21.05	0
3	Sharing of towel	Patients students	1.9	0.168
		Patients employees	0.319	0.572
4	Sharing of shaving razors/machine	Patients students	19.9	0
		Patients employees	1.39	0.238
5	Sharing of cosmetics	Patients students	32.55	0
		Patients employees	0.19	0.892

Fisher’s exact value was given for the variables not meeting the chi-square assumption of “expected count not less than 5 in each cell.” Alpha criterion = 0.05.

### 3.5 Prevalence and association of behavioral-related hepatitis risk factors

Assessment of behavioral-related factors shows that, amongst hepatitis- positive participants, smoking was reported in 8% of students and 10% of employees, while among hepatitis-negative participants smoking was observed in 7% of students and 9% of employees and was not found to have a relation with hepatitis (*p*-value > 0.5). Analysis of reusing the used syringes displays that 77% of hepatitis positive students, 79% of hepatitis negative students, 73% of hepatitis positive employees, and 69% of hepatitis negative employees made sure to use new syringes. The *X^2^* calculation shows it to be associated with students (48.3%, *p*-value 0.00). Among hepatitis-positive participants, a total of 56% (students) and 46% (employees) share their towels while among hepatitis-negative participants 57% of students and 47% of employees do the same, but this shows no significant association in either group [0.9, *p*-value 0.16 (students) and 0.3 *p*-value 0.57 (employees)].

Of the participants, 64% of hepatitis positive students, 56% of hepatitis negative students, 54% of hepatitis positive employees, and 50% of hepatitis negative employees share shaving razors/machines and this was found to be significant (19.9, *p*-value 0.00) in the student group.

Whereas, amongst females, 17% of hepatitis-positive students, 32% of hepatitis-negative students, 15% of hepatitis-positive employees, and 16% of hepatitis-negative students were reported to share their cosmetics. The *X^2^* test’s value was shown to be 32.5 (*p*-value 0.00) in the students group (Table 2, Figures 5, 6, and Supplementary Tables S1, S2).

### 3.6 Independent variables association with hepatitis by binary logistic regression

To explore the extent of risk factors’ influence on hepatitis, variables were analyzed individually by logistic regression. Multicollinearity assumptions were tested and found to be satisfied for binary logistic regression by all variables, i.e., tolerance value was greater than 0.1 and variance inflation factor (VIF) value was less than 5 and, hence, non-collinearity was detected among variables (Supplementary Tables S4, S5). All variables in the equation were shown in Supplementary Tables S6, S7.

Logistic regression analysis depicted different predictor variables to be linked with each group. In students, gender (male) with an adjusted odd ratio (AOR = 2.102 [95% CI = 1.62–2.71]), sharing shaving razors (AOR = 1.41, [95% CI = 1.14–1.76]), and vaccination (AOR = 1.87, [95% CI = 1.44–2.44]) was positively associated with hepatitis. However, income above 50,000 (AOR = 0.506, [95% CI = 0.334–0.745]), below 50,000 (AOR = 0.71,[95% CI = 0.511–0.987]), marital status (AOR = 0.451, [95% CI = 0.278–0.732]), dental treatment (AOR = 0.744, [95% CI = 0.558–0.991]), facial treatment (AOR = 0.532, [95% CI = 0.320–0.883]), and cosmetics sharing (AOR = 0.547, [95% CI = 0.425–0.703]) were found to be negatively linked with the likelihood of hepatitis in students (Table 3).

**Table 3:** Independent risk factors significantly associated with hepatitis (students group).

Risk factors	B	S.E.	Wald	df^a^	Sig.	EXP(B) (AOR^b^)	95% C.I. for EXP(B)	
							Lower	Upper
Socio-economic related factors								
Gender (Male)	0.743	0.131	32.094	1	0.00	2.102	1.625	2.718
Income			12.085	3	0.007			
Above 50,000	−0.681	0.197	11.956	1	0.001	0.506	0.344	0.745
Below 50,000	−0.342	0.168	4.156	1	0.04	0.710	0.511	0.987
Marital status	−0.796	0.247	10.390	1	0.001	0.451	0.278	0.732
Medical related factors								
Dental treatment	−0.296	0.147	4.075	1	0.044	0.744	0.558	0.991
Facial treatment	−0.631	0.259	5.948	1	0.015	0.532	0.320	0.883
Already vaccinated	0.630	0.134	21.991	1	0.000	1.877	1.443	2.442
Behavioral related risk factors								
Sharing shave razors/machines	0.350	0.110	10.148	1	0.001	1.419	1.144	1.761
Sharing cosmetics	−0.604	0.129	22.029	1	0.000	0.547	0.425	0.703

Reference category was chosen to be 1st. Sig., Significant values (*p*-values < 0.05); ^a^ df, Degree of freedom; ^b^ AOR, Adjusted odd ratio.

In employees, age group of 25–40 years (AOR = 4.406, [95% CI = 2.094–9.286]), 41–50 years (AOR = 4.721, [95% CI = 2.118–10.523]), above 50 years (AOR = 3.393, [95% CI = 1.407–8.185]), primary education (AOR = 2.313, [95% CI = 1.114–4.803]), household contacts (AOR = 2.013, [95% CI = 1.054–3.844]), surgery (AOR = 1.653, [95% CI = 1.086–2.514]), and vaccination (AOR = 2.331, [95% CI = 1.408–3.859]) were associated with a positive likelihood of hepatitis (Table 4).

**Table 4:** Independent risk factors significantly associated with hepatitis (employees group).

Risk factors	B	S.E.	Wald	df^a^	Sig.	EXP(B) (AOR^b^)	95% C.I. for EXP(B)	
							Lower	Upper
Age			17.063	3	0.001			
25–40 years	1.483	0.379	15.274	1	0.00	4.406	2.094	9.268
41–50 years	1.552	0.409	14.403	1	0.00	4.721	2.118	10.523
Above 50 years	1.222	0.449	7.394	1	0.007	3.393	1.407	8.185
Education			11.685	4	0.02			
Primary	0.838	0.373	5.057	1	0.02	2.313	1.114	4.803
Household contact	0.699	0.33	4.49	1	0.034	2.013	1.054	3.844
Surgery	0.502	0.214	5.503	1	0.019	1.653	1.086	2.514
Vaccination	0.846	0.257	10.814	1	0.001	2.331	1.408	3.859

Reference category was chosen to be 1st. Sig., Significant values (*p*-values < 0.05); ^a^df, Degree of freedom; ^b^ AOR, Adjusted odd ratio.

### 3.7 Lifestyle quality assessment of hepatitis-positive participants

The evaluation of quality lifestyle habits reveals that 50% of hepatitis-positive students and 40% of hepatitis-positive employees used to do exercise on a daily basis. Studying the food type prevalence, we observed normal food eaters represent high frequency in both the studied groups, i.e., 75% in hepatitis-positive students, 69% in hepatitis-negative students, 84% in hepatitis-positive employees, and 76% in hepatitis-negative employees in comparison to vegetarian, fast food, and meat eaters. Many hepatitis-positive participants, students (27%) and employees (57%), were noted to not go outdoor dining. A total of 36% of hepatitis-positive students, 45% of hepatitis-negative students, 36% of hepatitis-positive employees, and 39% of hepatitis-negative employees were spotted to be in the habit of eating fruits sliced by shopkeepers. The frequency of using filtered water in hepatitis-positive participants was noticed to be 51% (students) and 44% (employees). Studying the hygiene-related factors, it was observed that among hepatitis-positive participants 17% of students and 19% of employees did not wash their hands before eating, while in hepatitis-negative participants, 15% of students and 17% of employees did the same (Supplementary Table S8).

## 4 Discussion

Hepatitis is a contagious viral disease and different environmental and behavioral risk factors are known to contribute to its spread (24). The current study was conducted to monitor the prevalence and association of possible risk factors of hepatitis among studied students and employees. Our findings revealed that, among studied participants, HCV is more prevalent than HBV. A very small percentage of students suffered from both HCV and HBV infection. A previous study conducted in Sindh, Pakistan reported higher frequency (14.3%) of HCV than HBV (14.3%) (15). Another study reported a higher HCV prevalence (42.7%) than HBV (8.4%) in Punjab, Pakistan (25). A high frequency of 9.9% of HBV in comparison to 4.1% of HCV was noted in the Taiwan population (26).

A high prevalence of HBV was reported among male students and employees; among female students and employees, HCV was more prevalent, but an association was found with males as they were more at risk of suffering from hepatitis by a factor of 2.102. These findings were in agreement with an earlier study of Pakistan representing men to be more likely to suffer from hepatitis B, increasing its chance over 2.1 times, while women were 1.3 times more at risk of having HCV (25). In a previous study in the USA, male sex was indicated as a risk factor for hepatitis C with an odd ratio of 1.25 (95% CI 1.03, 1.51) (27). In our study, the independent association of hepatitis strain with any risk factor was not determined but overall our findings suggest that hepatitis was seen to be prevalent in the 16–25 years’ age group in students and in the 26–40 year age group in employees, which was in agreement with a previous survey that also shows HBV is more frequent in the age group of 16–30 years, while HCV was noted to be more prevalent in patients above 61 years (25). Age was noted to be associated with hepatitis among the employee group. Independent variable analysis of employees showed that the age group 25–40 years increases the likelihood of hepatitis by 4.406 factors. Similarly, the increase in age to 41–50 years also elevates the hepatitis risk by 4.721, and age above 50 contributed to an increase in its risk by 3.39 degrees. Our results were in agreement with a previous report suggesting an increase in age to be [OR 1.26 (95% CI 1.23, 1.30)] related to hepatitis (27). Khan et al. indicated increasing age as a risk factor for HBV and HCV with a respective odd ratio of 4.2 and 56.5. These findings suggested that, with increasing age, the chance of developing hepatitis will be increased, and more precautions are required for prevention. It was observed that, in students, hepatitis was more prevalent in graduates while in employees its rate of occurrence decreases with an increase in the literacy level as only the employees educated to a primary level were found to be hepatitis positive. But no independent education variable was found to be associated with hepatitis in student groups. A previous survey mentioned a high prevalence of HCV in men with a primary education and graduates (24). According to a previous study in Pakistan, no association with education was observed (10). Social status was also noted, showing that its frequency was high in students and employees belonging to middle-class and low-earning families and contributed to hepatitis prevalence. In students, low income increases the hepatitis likelihood by an extent of 0.710. High income decreases its risk by 0.506 but no association was noted in hepatitis-positive participants (employee) and an independent income variable. Previously, unemployment was described to be correlated with HCV prevalence, which supports our result (28). Here, literacy shows a link with hepatitis as education provides knowledge about the disease and in Pakistan high prevalence of the disease is majorly due to illiteracy and unemployment. The percentage of married students and employees was low, with a high frequency of intrafamily marriage, and they were less likely to be exposed to hepatitis infection by 4.51 factors. High frequency (46.7%) of HCV in a single individual was also reported earlier (24). It was observed that, as compared to hepatitis negative-participants, a high percentage of household contacts was reported by hepatitis- positive participants, showing that hepatitis is a contagious disease and spread by contact with other members and was also found to be associated with hepatitis by *X*^2^ test. It was noted that, in employees, the hepatitis risk increases 2.01 times due to household contact. In our study, a very small frequency of infected mothers of hepatitis positive participants was noted, which shows that in our population mother-to-child transmission of infection is not common.

Several medical-related risk factors were also noted to be linked with hepatitis. In the present study, it was seen that students and employees who were vaccinated against hepatitis have a higher frequency of infection in comparison to hepatitis-negative participants. No association between hepatitis, blood group, and blood transfusion was noted, and their prevalence was more in the hepatitis-negative participants. Previously, contradictions were noticed in studies exploring the role of blood groups as hepatitis risk factors. In Pakistan, in an earlier study, association of blood group A with HBV was noted. Moreover, blood group O was reported to be protective against HBV (29). In 2021, a study conducted in Multan, Pakistan reported blood group O to be a risk factor for HCV (30). Further meta-analysis demonstrated a lower likelihood of HBV in blood group B individuals (31). Formerly, blood transfusion frequency was also noted to be higher in the hepatitis-negative participants but was found to be a risk factor for HBV with an odd ratio of 7.03 (CI = 3.37–14.66) and HCV with 14.88 (CI = 8.14–27.16) odd ratio (15). In Georgia, blood transfusion was considered as (AOR = 4.5, 95% CI = 2.8, 7.2) a HCV risk factor (28). In comparison to hepatitis-negative students, hepatitis-positive students were observed to have facial treatments, which was found to be not associated as shown by the *X*^2^ test, but it was found to be less likely linked with hepatitis by 0.525 factors. Dental treatment frequency was higher in hepatitis-positive employees in contrast to the hepatitis-negative participants. By the logistic model, the significant independent association of dental treatment was recorded to be 0.744, indicating that students having dental treatment were less likely to have hepatitis than those without treatment. Hepatitis-positive participants represent a higher percentage of surgery than hepatitis-negative participants and were significantly associated with hepatitis as reported by the *X*^2^ test. These results were similar to the previous one where surgery and dental treatment were described to be associated with hepatitis by a respective odd factor of 2.7 and 4.1 (10). An earlier study stated surgery and dental treatment as not being hepatitis risk factors (24). Facial treatment was found to be less likely to be associated with hepatitis by a factor of 5.32 in the student group as its frequency was noted to be higher among hepatitis-negative participants. We have not found any intravenous drug abuse among our participants, however addiction to other orally taken drugs was noted to be significant among hepatitis-positive student participants. The association of vaccination and hepatitis was found with more likelihood (1.87) of hepatitis in vaccinated students and employees (2.31). These are the controversial results as a decline in HBV was narrated by the use of vaccination in China (32). A high percentage of infection in already vaccinated hepatitis-positive participants might be due to the immunosuppressant participants, as hepatitis vaccines are known internationally to lower the infection risk.

Various kinds of behavioral-related factors were evaluated in the present study and found to be associated with hepatitis. Smoking frequency did not vary much among hepatitis-positive and -negative participants and was found not to be a hepatitis risk factor. A previous study also described smoking to not be related to chronic hepatitis B (33). In our study, utilization of used syringes was found to be associated with hepatitis by *X*^2^-test but its independent association with hepatitis cannot be recorded by logistic regression analysis. However, previous research conducted in Pakistan showed reused syringes to be a hepatitis risk factor (34). Hygiene is the main factor to be considered to avoid contracting the hepatitis virus. Exploration of personal hygiene-related factors depicted the association of hepatitis with two factors: sharing shaving razors and machines. The frequency of sharing shaving blades was higher in the hepatitis-positive participants and is reported to increase the hepatitis risk by 1.41 factors in students sharing their shaving razors and machines. In earlier studies, shaving at a barber was found to be a hepatitis risk factor in Pakistan (25). As the cosmetic sharing percentage was higher in hepatitis-negative participants, it was found to be negatively linked with hepatitis by a factor of 0.547. This indicates that unhygienic measurements can be considered a way for hepatitis to spread.

Further assessment of the quality of lifestyle done by comparing the lifestyle-related factors among hepatitis-positive and -negative participants depicted that the studied hepatitis-positive participants have adopted a healthy lifestyle which will be necessary to reduce further damage to the liver and the progression of infection. It was observed that, in the study participants, a large number of hepatitis-positive participants have a habit of exercising, eating normal food, avoiding outside dining, and using filtered water. All these factors are required for combating infection. These will also help in preventing exposure to other hepatitis strains. The only drawback is that the hepatitis-positive participants did not care about proper hand washing, which is crucial, as hand washing is helpful in preventing the spread of infection.

Although this study has proven to be successful in making IUB the first hepatitis-free university, certain limitations were there in exploring the information regarding the prevalence and hepatitis-associated factors. This research was limited to IUB students and employees and did not target any specific ethnic group or population of a specific province or district. Therefore, this is generalized research and does not define the association of hepatitis risk factors with any specific ethnic group or population of a specific province or district of the country.

## 5 Conclusion

Many hepatitis-related risk factors have been explored by the described research and can help design preventive strategies for hepatitis. We have recognized that socioeconomic parameters, medical-related factors, and behavioral measures are associated with a high prevalence of hepatitis in both students and employees. As the lifestyle of students differs from employees, risk factors association also varied accordingly. An ample understanding of risk factors can pave the way for better screening and prevention measurements as hepatitis is a preventable disease. The effective implementation of this awareness, screening, and vaccination program conferred IUB the status of Pakistan’s first Hepatitis-free university and, to continue this project (to keep IUB hepatitis-free), hepatitis screening and vaccination has been made compulsory for every admitted student. To ensure the successful implementation of the vaccination facility, the hepatitis vaccination center has been established in the IUB medical division. In the future, the identified risk factors will help raise awareness about hepatitis and reduce its prevalence in the Pakistani population. This study has provided general guidelines to be followed for other institutions and organizations, in Pakistan and abroad, to make them hepatitis-free in particular or any other infectious disease in general.

## Data availability statement

The original contributions presented in the study are included in the article/Supplementary material, further inquiries can be directed to the corresponding author.

## Ethics statement

The studies involving humans were approved by Hepatitis Control Program Core Committee, The Islamia University of Bahawalpur, Pakistan. The studies were conducted in accordance with the local legislation and institutional requirements. The participants provided their written informed consent to participate in this study. Written informed consent was obtained from the individual(s) for the publication of any potentially identifiable images or data included in this article.

## Author contributions

SE: Conceptualization, Investigation, Project administration, Supervision, Validation, Writing – review & editing. IA: Data curation, Formal analysis, Investigation, Methodology, Software, Validation, Writing – original draft. WM: Data curation, Formal analysis, Investigation, Methodology, Writing – original draft. SA: Project administration, Writing – review & editing. MA: Project administration, Writing – review & editing. NA: Conceptualization, Project administration. AK: Validation, Supervision. YH: Data curation, Writing – original draft. MU: Data-curation. UC: Supervision, Validation. SS: Project administration.
