# Supplementary material for: Screening of hepatitis B and C viral infection, recognition of risk factors, and immunization of patients against hepatitis B virus: a module developed for effective hepatitis control
Source: Front Public Health. 2023 Nov 23;11:1269209. doi: 10.3389/fpubh.2023.1269209 (PMC10721974; doi:10.3389/fpubh.2023.1269209)
Supplement: Supplementary file 1 [file Table_1.docx]

**S1 Table :** **Frequency of medical associated risk factors in hepatitis negative and positive participants**

| **Sr.No.** | **Factors** | **Response** | **Hepatitis negative students** | | **Hepatitis positive Students** | | **Sr.No.** | **Factors** | **Response** | **Hepatitis negative employees** | | **Hepatitis positive employees** | |  |
| --- | --- | --- | --- | --- | --- | --- | --- | --- | --- | --- | --- | --- | --- | --- |
|  |  |  | **Numbers** | **Frequency** | **Numbers** | **Frequency** |  |  |  | **Numbers** | **Frequency** | **Number** | **Frequency** | |
| 1 | Blood Group | A+ | 1296 | 12% | 40 | 8% | 1 | Blood Group | A+ | 181 | 10% | 9 | 5% | |
|  |  | A- | 93 | 1% | 2 | 0% |  |  | A- | 18 | 1% | 0 | 0% | |
|  |  | B+ | 2542 | 24% | 94 | 20% |  |  | B+ | 300 | 17% | 28 | 15% | |
|  |  | B- | 168 | 2% | 6 | 1% |  |  | B- | 30 | 2% | 1 | 1% | |
|  |  | AB+ | 582 | 6% | 20 | 4% |  |  | AB+ | 86 | 5% | 3 | 2% | |
|  |  | AB- | 46 | 0% | 1 | 0% |  |  | AB- | 5 | 0% | 0 | 0% | |
|  |  | O+ | 1657 | 16% | 60 | 13% |  |  | O+ | 249 | 14% | 12 | 6% | |
|  |  | O- | 229 | 2% | 7 | 1% |  |  | O- | 21 | 1% | 4 | 2% | |
|  |  | Not Answered | 3857 | 37% | 248 | 52% |  |  | Not Answered | 893 | 50% | 127 | 69% | |
| 2 | Blood Transfusion History | Yes | 1502 | 14% | 65 | 14% | 2 | Blood Transfusion History | Yes | 305 | 17% | 32 | 17% | |
|  |  | No | 8968 | 86% | 402 | 84% |  |  | No | 1478 | 83% | 151 | 82% | |
|  |  | 1-2 | 1104 | 11% | 46 | 10% |  |  | 1-2 | 196 | 11% | 23 | 12% | |
|  |  | 3-4 | 229 | 2% | 8 | 2% |  |  | 3-4 | 55 | 3% | 2 | 1% | |
|  |  | 5 or above | 169 | 2% | 11 | 2% |  |  | 5 or above | 54 | 3% | 7 | 4% | |
|  |  | Not Answered | 0 | 0% | 11 | 2% |  |  | Not Answered | 0 | 0% | 1 | 1% | |
| 3 | Dental Treatment History | Yes | 1620 | 15% | 56 | 12% | 3 | Dental Treatment History | Yes | 319 | 18% | 24 | 13% | |
|  |  | No | 8850 | 85% | 404 | 85% |  |  | No | 1464 | 82% | 155 | 84% | |
|  |  | 1-2 | 1312 | 13% | 40 | 8% |  |  | 1-2 | 241 | 14% | 16 | 9% | |
|  |  | 3-4 | 190 | 2% | 10 | 2% |  |  | 3-4 | 41 | 2% | 5 | 3% | |
|  |  | 5 or above | 118 | 1% | 6 | 1% |  |  | 5 or above | 37 | 2% | 3 | 2% | |
|  |  | Not Answered | 0 | 0% | 18 | 4% |  |  | Not  Answered | | 0% | 0 | 0% | |
| 4 | Facial Treatment History | Yes | 653 | 6% | 16 | 3% | 4 | Facial Treatment History | Yes | 44 | 2% | 2 | 1% | |
|  |  | No | 9817 | 94% | 462 | 97% |  |  | No | 1739 | 98% | 182 | 98% | |
|  |  | 1-2 | 490 | 5% | 14 | 3% |  |  | 1-2 | 32 | 2% | 1 | 1% | |
|  |  | 3-4 | 90 | 1% | 2 | 0% |  |  | 3-4 | 3 | 0% | 0 | 0% | |
|  |  | 5 or above | 73 | 1% | 0 | 0% |  |  | 5 or above | 8 | 0% | 1 | 1% | |
|  |  | Not Answered | 0 | 0% | 0 | 0% |  |  | Not Answered | 0 | 0% | 0 | 0% | |
| 5 | Surgery History | Yes | 770 | 7% | 42 | 9% | 5 | Surgery History | Yes | 216 | 12% | 32 | 17% | |
|  |  | No | 9700 | 93% | 436 | 91% |  |  | No | 1567 | 88% | 152 | 82% | |
|  |  | 1-2 | 704 | 7% | 37 | 8% |  |  | 1-2 | 177 | 10% | 29 | 16% | |
|  |  | 3-4 | 51 | 0% | 4 | 1% |  |  | 3-4 | 28 | 2% | 3 | 2% | |
|  |  | 5 or above | 15 | 0% | 1 | 0% |  |  | 5 or above | 11 | 1% | 0 | 0% | |
|  |  | Not Answered | 0 | 0% | 0 | 0% |  |  | Not Answered | 0 | 0% | 0 | 0% | |
| 6 | Addiction to Any Drug | Yes | 13 | 0.12% | 1 | 0.21% | 6 | Addiction to Any Drug | Yes | 1 | 0% | 0 | 0% | |
|  |  | No | 10457 | 99.88% | 477 | 99.79% |  |  | No | 1782 | 100% | 184 | 99% | |
|  |  | Not Answered | 0 | 0% | 0 | 0% |  |  | Not Answered | 0 | 0% | 0 | 0% | |
| 7 | Vaccinated against HBV | Yes | 959 | 9% | 72 | 15% | 7 | Vaccinated against HBV | Yes | 95 | 5% | 22 | 12% | |
|  |  | No | 9511 | 91% | 392 | 82% |  |  | No | 1688 | 95% | 161 | 87% | |
|  |  | Not Answered | 0 | 0% | 14 | 3% |  |  | Not Answered | 0 | 0% | 1 | 1% | |

**S2 Table: Frequency of behavioral associated risk factors in hepatitis negative and positive participants**

| **Sr.No.** | **Factors** | **Response** | **Hepatitis negative students** | | **Hepatitis positive Students** | | **Sr.No.** | **Factors** | **Response** | **Hepatitis negative employees** | | **Hepatitis positive employees** | |
| --- | --- | --- | --- | --- | --- | --- | --- | --- | --- | --- | --- | --- | --- |
|  |  |  | **Numbers** | **Frequency** | **Numbers** | **Frequency** |  |  |  | **Numbers** | **Frequency** | **Number** | **Frequency** |
| 1 | Smoking | Yes | 758 | 7% | 38 | 8% | 1 | Smoking | Yes | 154 | 9% | 18 | 10% |
|  |  | No | 9712 | 93% | 440 | 92% |  |  | No | 1629 | 91% | 166 | 90% |
|  |  | Not Answered | 0 | 0% | 0 | 0% |  |  | Not Answered | 0 | 0% | 0 | 0% |
| 2 | Avoid reused syringe | Yes | 8221 | 79% | 366 | 77% | 3 | Avoid reused syringe | Yes | 1230 | 69% | 135 | 73% |
|  |  | No | 2249 | 21% | 98 | 21% |  |  | No | 553 | 31% | 48 | 26% |
|  |  | Not Answered | 0 | 0% | 14 | 3% |  |  | Not Answered | 0 | 0% | 1 | 1% |
| 3 | Sharing towel | Yes | 5940 | 57% | 266 | 56% | 3 | Sharing towel | Yes | 841 | 47% | 85 | 46% |
|  |  | No | 4530 | 43% | 177 | 37% |  |  | No | 942 | 53% | 87 | 47% |
|  |  | Not Answered | 0 | 0% | 35 | 7% |  |  | Not Answered | 0 | 0% | 12 | 6% |
| 4 | Sharing Shaving razors/machine | Yes | 5816 | 56% | 306 | 64% | 4 | Sharing Shaving Blade | Yes | 883 | 50% | 99 | 54% |
|  |  | No | 4654 | 44% | 157 | 33% |  |  | No | 900 | 50% | 84 | 45% |
|  |  | Not Answered | 0 | 0% | 15 | 3% |  |  | Not Answered | 0 | 0% | 1 | 1% |
| 5 | Sharing of cosmetics | Yes | 3302 | 32% | 83 | 17% | 5 | Sharing of cosmetics | Yes | 287 | 16% | 27 | 15% |
|  |  | No | 7168 | 68% | 360 | 75% |  |  | No | 1496 | 84% | 145 | 78% |
|  |  | Not Answered | 0 | 0% | 35 | 7% |  |  | Not Answered | 0 | 0% | 12 | 6% |

**S3 Table. Association of socioeconomic and demographic parameters with hepatitis**

| **Sr.**  **No** | **Risk factor** | **Groups** | ***X^2^* Values** | ***P*-value** | **Sr.**  **No.** | **Family History** | **Groups** | ***X^2^* Values** | ***P*-value** |
| --- | --- | --- | --- | --- | --- | --- | --- | --- | --- |
|  | **Demographic Parameters** |  |  |  |  |  |  |  |  |
| 1 | Gender | Patients Students | 94.59 | 0 | 5 | Marital status | Patients Students | 291.6 | 0 |
|  |  | Patients Employees | 1.26 | .26 |  |  | Patients Employees | 98.1 | 0 |
| 2 | Age | Patients Students | 6.36 | .09 | 6 | Household contact | Patients Students | 45.27 | 0 (Fisher exact value 0.01) |
|  |  | Patients Employees | 17.08 | .001 |  |  | Patients Employees | 2.59 | 0.107 |
| 3 | Education | Patients Students | 2.76 | .59 | 7 | Hepatitis history in relative | Patients Students | 8.688 | 0 |
|  |  | Patients Employees | 19 | 0 |  |  | Patients Employees | 0.761 | 0.383 (Fisher value.311) |
| 4 | Income | Patients Students | 17.35 | .001 | 8 | Marriage trend | Patients Students | 38.05 | 0 |
|  |  | Patients Employees | 7.27 | .06 |  |  | Patients Employees | 7.967 | 0.005 |

Fisher exact value was given for the variables not meeting the chi-square assumption of “expected count not less than 5 in each cell”. Alpha criterion =0.05.

**S4 Table. Multicollinearity test of students predictor variables for binary logistic regression**

| **Coefficients^a^** | | | | | | | | | |
| --- | --- | --- | --- | --- | --- | --- | --- | --- | --- |
| **Model** | **Unstandardized Coefficients** | | **Standardized Coefficients** | **t** | **Sig.** | **95.0% Confidence Interval for B** | | **Collinearity Statistics** | |
|  | **B** | **Std. Error** | **Beta** |  |  | **Lower Bound** | **Upper Bound** | **Tolerance** | **VIF** |
| (Constant) | 1.599 | .339 |  | 4.720 | .000 | .934 | 2.264 |  |  |
| Gender | .084 | .028 | .156 | 3.029 | .003 | .030 | .138 | .452 | 2.214 |
| Age | -.057 | .032 | -.081 | -1.788 | .074 | -.120 | .006 | .579 | 1.727 |
| Education | -.009 | .009 | -.036 | -1.021 | .308 | -.027 | .009 | .979 | 1.021 |
| Income | .013 | .009 | .058 | 1.496 | .135 | -.004 | .031 | .808 | 1.238 |
| Marital status | -.094 | .045 | -.090 | -2.086 | .037 | -.182 | -.006 | .641 | 1.561 |
| Relative Hepatitis Historty | .021 | .085 | .008 | .243 | .808 | -.147 | .188 | .985 | 1.015 |
| Marriage Trend | -.023 | .019 | -.042 | -1.183 | .237 | -.060 | .015 | .967 | 1.034 |
| Mother hepatitis positive | .000 | .006 | -.001 | -.028 | .977 | -.011 | .011 | .941 | 1.063 |
| Blood Transfusion | -.045 | .026 | -.063 | -1.698 | .090 | -.096 | .007 | .872 | 1.147 |
| Dental Treatment | -.008 | .024 | -.012 | -.336 | .737 | -.056 | .040 | .946 | 1.057 |
| Facial Treatment | -.021 | .037 | -.021 | -.583 | .560 | -.093 | .050 | .967 | 1.034 |
| Surgery | -.007 | .037 | -.007 | -.192 | .848 | -.080 | .065 | .962 | 1.040 |
| Drug addiction | -.104 | .191 | -.019 | -.542 | .588 | -.479 | .272 | .972 | 1.029 |
| Already vaccinated | .055 | .027 | .072 | 2.050 | .041 | .002 | .109 | .980 | 1.021 |
| Smoking | .007 | .040 | .006 | .169 | .866 | -.072 | .085 | .925 | 1.081 |
| Reused syringe | -.008 | .013 | -.021 | -.600 | .549 | -.032 | .017 | .961 | 1.040 |
| Share shave razor/machine | .003 | .021 | .006 | .147 | .883 | -.037 | .043 | .847 | 1.180 |
| Share towel | -.005 | .019 | -.010 | -.281 | .779 | -.043 | .032 | .970 | 1.031 |
| Share cosmetics | .019 | .027 | .033 | .683 | .495 | -.035 | .072 | .507 | 1.971 |

^a^ Dependent Variable: Student hepatitis

**S5 Table.** **Multicollinearity test of employees predictor variables for binary logistic regression**

| **Coefficients^a^** | | | | | | | | | |
| --- | --- | --- | --- | --- | --- | --- | --- | --- | --- |
| **Model** | **Unstandardized Coefficients** | | **Standardized Coefficients** | **t** | **Sig.** | **95.0% Confidence Interval for B** | | **Collinearity Statistics** | |
|  | **B** | **Std. Error** | **Beta** |  |  | **Lower Bound** | **Upper Bound** | **Tolerance** | **VIF** |
| (Constant) | 1.285 | 1.046 |  | 1.229 | .224 | -.810 | 3.380 |  |  |
| Gender | -.162 | .133 | -.244 | -1.220 | .228 | -.428 | .104 | .352 | 2.841 |
| Age | .104 | .071 | .275 | 1.469 | .147 | -.038 | .246 | .401 | 2.495 |
| Education | .026 | .029 | .131 | .883 | .381 | -.033 | .085 | .636 | 1.571 |
| Income | -.057 | .040 | -.212 | -1.410 | .164 | -.137 | .024 | .619 | 1.616 |
| Marital status | .055 | .065 | .165 | .858 | .395 | -.074 | .185 | .377 | 2.653 |
| Relative Hepatitis Historty | -.096 | .226 | -.058 | -.427 | .671 | -.548 | .355 | .759 | 1.318 |
| Marriage Trend | -.165 | .100 | -.254 | -1.660 | .102 | -.365 | .034 | .597 | 1.675 |
| Mother hepatitis positive | -.024 | .033 | -.121 | -.716 | .477 | -.091 | .043 | .494 | 2.022 |
| Blood Transfusion | .021 | .108 | .028 | .194 | .847 | -.196 | .238 | .690 | 1.448 |
| Dental Treatment | .060 | .119 | .070 | .509 | .613 | -.177 | .298 | .733 | 1.365 |
| Facial Treatment | .255 | .266 | .126 | .958 | .342 | -.278 | .788 | .806 | 1.241 |
| Surgery | -.081 | .163 | -.077 | -.495 | .622 | -.407 | .246 | .586 | 1.707 |
| Already vaccinated | .008 | .122 | .008 | .065 | .949 | -.237 | .253 | .858 | 1.165 |
| Smoking | -.123 | .123 | -.139 | -1.001 | .321 | -.369 | .123 | .729 | 1.372 |
| Reused syringe | .034 | .055 | .086 | .616 | .541 | -.077 | .144 | .724 | 1.381 |
| Share shave razor/machine | .102 | .099 | .158 | 1.036 | .305 | -.096 | .301 | .600 | 1.665 |
| Share towel | -.009 | .099 | -.015 | -.096 | .924 | -.207 | .188 | .603 | 1.659 |
| Share cosmetics | -.139 | .137 | -.175 | -1.011 | .316 | -.414 | .136 | .467 | 2.140 |

^a^ Dependent variable=Hepatitis positive employees

Drug addiction does not fit model and thus not included.

**S6 Table. Variables in the equations (students group) by logistic regression**

| **Risk factors** | **B** | **S.E.** | **Wald** | **df^a^** | **Sig.** | **EXP(B) (AOR^b^ )** | | | **95% C.I. for EXP(B)** | |
| --- | --- | --- | --- | --- | --- | --- | --- | --- | --- | --- |
|  |  |  |  |  |  |  |  |  |  |  |
|  |  |  |  |  |  |  |  |  | **Lower** | **Upper** |
| **Socio-economic related factors** | | | | | | | | | | |
| Gender (Male) | 0.743 | 0.131 | 32.094 | 1 | 0 | | 2.102 | | 1.625 | 2.718 |
| Age |  |  | 0.52 | 3 | 0.914 | |  | |  |  |
| 25-40 years | -0.123 | 0.262 | 0.219 | 1 | 0.639 | | 0.885 | | 0.53 | 1.477 |
| 41-50 years | -0.317 | 0.465 | 0.465 | 1 | 0.495 | | 0.728 | | 0.293 | 1.812 |
| Above 50 | -0.155 | 0.661 | 0.055 | 1 | 0.814 | | 0.856 | | 0.234 | 3.128 |
| Income |  |  | 12.085 | 3 | 0.007 | |  | |  |  |
| Above 20000 | -0.399 | 0.226 | 3.117 | 1 | 0.077 | | 0.671 | | 0.431 | 1.045 |
| Above 50,000 | -0.681 | 0.197 | 11.956 | 1 | 0.001 | | 0.506 | | 0.344 | 0.745 |
| Below 50,000 | -0.342 | 0.168 | 4.156 | 1 | 0.041 | | 0.71 | | 0.511 | 0.987 |
| Education |  |  | 3.078 | 4 | 0.545 | |  | |  |  |
| Intermediate | -0.269 | 0.217 | 1.54 | 1 | 0.215 | | 0.764 | | 0.5 | 1.169 |
| Matric | -0.642 | 0.619 | 1.076 | 1 | 0.3 | | 0.526 | | 0.156 | 1.771 |
| Post Graduate | 0.043 | 0.179 | 0.059 | 1 | 0.808 | | 1.044 | | 0.735 | 1.483 |
| Primary | 0.201 | 0.506 | 0.159 | 1 | 0.69 | | 1.223 | | 0.454 | 3.294 |
| Marital status | -0.796 | 0.247 | 10.39 | 1 | 0.001 | | 0.451 | | 0.278 | 0.732 |
| Household contact | .231 | 1.059 | .048 | 1 | .827 | | 1.260 | | .158 | 10.036 |
| Relative hepatitis | -.185 | .258 | .514 | 1 | .474 | | .831 | | .501 | 1.378 |
| Mother infected with hepatitis | .042 | .075 | .312 | 1 | .576 | | 1.043 | | .900 | 1.208 |
| Constant | -2.004 | 1.378 | 2.114 | 1 | .146 | | .135 | |  |  |
| **Medical related risk factors** | | | | | | | | | | |
| Blood transfusion | -.028 | .138 | .040 | 1 | .842 | | | .973 | .742 | 1.276 |
| Dental treatment | -.296 | .147 | 4.075 | 1 | .044 | | | .744 | .558 | .991 |
| Facial treatment | -.631 | .259 | 5.948 | 1 | .015 | | | .532 | .320 | .883 |
| Surgery | .256 | .168 | 2.313 | 1 | .128 | | | 1.291 | .929 | 1.795 |
| Drug addiction | .632 | 1.043 | .367 | 1 | .545 | | | 1.881 | .244 | 14.519 |
| Already vaccinated | .630 | .134 | 21.991 | 1 | .000 | | | 1.877 | 1.443 | 2.442 |
| Constant | -4.079 | 1.151 | 12.567 | 1 | .000 | | | .017 |  |  |
| **Behavioral risk factors** |  |  |  |  |  | | |  |  |  |
| Smoker | -.087 | .180 | .233 | 1 | .629 | | | .917 | .644 | 1.305 |
| Reused syringe | -.006 | .063 | .009 | 1 | .926 | | | .994 | .878 | 1.126 |
| Sharing shave razors/machines | .350 | .110 | 10.148 | 1 | .001 | | | 1.419 | 1.144 | 1.761 |
| Towel sharing | .105 | .100 | 1.106 | 1 | .293 | | | 1.111 | .913 | 1.351 |
| Sharing cosmetics | -.604 | .129 | 22.029 | 1 | .000 | | | .547 | .425 | .703 |
| Constant | -2.186 | .494 | 19.545 | 1 | .000 | | | .112 |  |  |

Reference category is chosen to be 1^st^. Sig.= Significant values (p-values <0.05), ^a^ df=Degree of freedom,

^b^ AOR=Adjusted odd ratio.

**S7 Table. Variables in the Equation (employees group) by logistic regression.**

| **Risk Factors** | **B** | **S.E.** | **Wald** | **df^a^** | **Sig.** | **EXP(B) (AORb )** | **95% C.I.for EXP(B)** | |
| --- | --- | --- | --- | --- | --- | --- | --- | --- |
|  |  |  |  |  |  |  |  |  |
|  |  |  |  |  |  |  | **Lower** | **Upper** |
| **Socio-economic factors** | | | | | | | | |
| Gender (Male) | -0.36 | 0.229 | 2.469 | 1 | 0.116 | 0.698 | 0.445 | 1.093 |
| Age |  |  | 17.063 | 3 | 0.001 |  |  |  |
| 25-40 years | 1.483 | 0.379 | 15.274 | 1 | 0 | 4.406 | 2.094 | 9.268 |
| 41-50 years | 1.552 | 0.409 | 14.403 | 1 | 0 | 4.721 | 2.118 | 10.523 |
| Above 50 years | 1.222 | 0.449 | 7.394 | 1 | 0.007 | 3.393 | 1.407 | 8.185 |
| Education |  |  | 11.685 | 4 | 0.02 |  |  |  |
| Intermediate | -0.251 | 0.524 | 0.23 | 1 | 0.632 | 0.778 | 0.278 | 2.174 |
| Matric | 0.099 | 0.413 | 0.058 | 1 | 0.81 | 1.104 | 0.491 | 2.482 |
| Post Graduates | 0.088 | 0.563 | 0.024 | 1 | 0.876 | 1.092 | 0.362 | 3.29 |
| Primary | 0.838 | 0.373 | 5.057 | 1 | 0.025 | 2.313 | 1.114 | 4.803 |
| Income |  |  | 2.382 | 3 | 0.497 |  |  |  |
| Above 20,000 | 0.143 | 0.287 | 0.248 | 1 | 0.619 | 1.154 | 0.657 | 2.025 |
| Above 50,000 | -0.519 | 0.507 | 1.045 | 1 | 0.307 | 0.595 | 0.22 | 1.609 |
| Below 50,000 | -0.209 | 0.295 | 0.502 | 1 | 0.478 | 0.811 | 0.455 | 1.446 |
| Constant | -4 | 0.528 | 57.409 | 1 | 0 | 0.018 |  |  |
| Household contact | 0.699 | 0.33 | 4.49 | 1 | 0.034 | 2.013 | 1.054 | 3.844 |
| Relative hepatitis | 0.681 | 0.799 | 0.726 | 1 | 0.394 | 1.975 | 0.413 | 9.457 |
| Marriage trend (within family) | 0.037 | 0.207 | 0.032 | 1 | 0.858 | 1.038 | 0.691 | 1.558 |
| Constant | -2.774 | 0.172 | 258.817 | 1 | 0 | 0.062 |  |  |
| **Medical related risk factors** | | | | | | | | |
| Blood transfusion | -0.035 | 0.211 | 0.028 | 1 | 0.867 | 0.965 | 0.639 | 1.459 |
| Dental treatment | -0.423 | 0.232 | 3.314 | 1 | 0.069 | 0.655 | 0.415 | 1.033 |
| Facial treatment | -0.811 | 0.732 | 1.229 | 1 | 0.268 | 0.444 | 0.106 | 1.864 |
| Surgery | 0.502 | 0.214 | 5.503 | 1 | 0.019 | 1.653 | 1.086 | 2.514 |
| Vaccination | 0.846 | 0.257 | 10.814 | 1 | 0.001 | 2.331 | 1.408 | 3.859 |
| Constant | -2.644 | 0.314 | 70.937 | 1 | 0 | 0.071 |  |  |
| **Behavioral risk factors** | | | | | | | | |
| Smoker | .119 | .273 | .191 | 1 | .662 | 1.127 | .660 | 1.924 |
| Reused syringe | .035 | .095 | .135 | 1 | .714 | 1.035 | .860 | 1.247 |
| Sharing shave razors/machines | .147 | .174 | .709 | 1 | .400 | 1.158 | .823 | 1.629 |
| Towel sharing | .057 | .163 | .124 | 1 | .725 | 1.059 | .770 | 1.457 |
| Sharing cosmetics | .018 | .224 | .006 | 1 | .936 | 1.018 | .656 | 1.581 |
| Constant | -3.018 | .775 | 15.168 | 1 | .000 | .049 |  |  |

Reference category is chosen to be 1^st^. Sig.= Significant values (p-values <0.05). ^a^ df=Degree of freedom,

^b^ AOR=Adjusted odd ratio.

**S8 Table:** **Frequency of lifestyle quality assessment parameters in hepatitis negative and positive participants**

| **Sr.No.** | **Factors** | **Response** | **Hepatitis negative students** | | **Hepatitis positive Students** | | **Sr.No.** | **Factors** | **Response** | **Hepatitis negative employees** | | **Hepatitis positive employees** | |
| --- | --- | --- | --- | --- | --- | --- | --- | --- | --- | --- | --- | --- | --- |
|  |  |  | **Numbers** | **Frequency** | **Numbers** | **Frequency** |  |  |  | **Numbers** | **Frequency** | **Number** | **Frequency** |
| 1 | Exercise | Yes | 3975 | 38% | 238 | 50% | 1 | Exercise | Yes | 615 | 34% | 74 | 40% |
|  |  | No | 6495 | 62% | 226 | 47% |  |  | No | 1168 | 66% | 109 | 59% |
|  |  | Not Answered | 0 | 0% | 14 | 3% |  |  | Not Answered | 0 | 0% | 1 | 1% |
| 2 | Food Type | Normal Food | 7248 | 69% | 359 | 75% | 2 | Food Type | Normal Food | 1352 | 76% | 156 | 84% |
|  |  | Fast Food | 1255 | 12% | 27 | 6% |  |  | Fast Food | 62 | 3% | 4 | 2% |
|  |  | Meat | 582 | 6% | 22 | 5% |  |  | Meat | 52 | 3% | 3 | 2% |
|  |  | Vegetables | 1056 | 10% | 46 | 10% |  |  | Vegetables | 192 | 11% | 13 | 7% |
|  |  | Not Answered | 329 | 3% | 24 | 5% |  |  | Not Answered | 125 | 7% | 8 | 4% |
| 3 | Dine Out Side | Daily | 2036 | 19% | 91 | 19% | 3 | Dine Out Side | Daily | 100 | 6% | 16 | 9% |
|  |  | Weekly | 2774 | 26% | 88 | 18% |  |  | Weekly | 159 | 9% | 14 | 8% |
|  |  | Monthly | 2053 | 20% | 86 | 18% |  |  | Monthly | 217 | 12% | 23 | 12% |
|  |  | Rarely | 2828 | 27% | 157 | 33% |  |  | Rarely | 1009 | 57% | 110 | 59% |
|  |  | Not Answered | 779 | 7% | 56 | 12% |  |  | Not Answered | 298 | 17% | 21 | 11% |
| 4 | Filtered water | Yes | 6382 | 61% | 245 | 51% | 4 | Filtered water | Yes | 899 | 50% | 82 | 44% |
|  |  | No | 4088 | 39% | 219 | 46% |  |  | No | 884 | 50% | 101 | 55% |
|  |  | Not Answered | 0 | 0% | 14 | 3% |  |  | Not Answered | 0 | 0% | 1 | 1% |
| 3 | Hand washing | Yes | 8949 | 85% | 383 | 80% | 3 | Hand washing | Yes | 1471 | 83% | 148 | 80% |
|  |  | No | 1521 | 15% | 81 | 17% |  |  | No | 312 | 17% | 35 | 19% |
|  |  | Not Answered | 0 | 0% | 14 | 3% |  |  | Not Answered | 0 | 0% | 1 | 1% |
